# What works for whom: a systematic review of inequalities in inclusion and effectiveness of social interventions for mental ill- health

**DOI:** 10.1007/s00127-025-02984-3

**Published:** 2025-09-22

**Authors:** Anna Greenburgh, Helen Baldwin, Hannah Weir, Zara Asif, Dionne Laporte, Mark Bertram, Achille Crawford, Gabrielle Duberry, Shoshana Lauter, Brynmor Lloyd-Evans, Cassandra Lovelock, Jayati Das-Munshi, Craig Morgan

**Affiliations:** 1 Institute of Psychiatry, Psychology & Neuroscience, King’s College London, London, UK; 2 ESRC Centre for Society and Mental Health, King’s College London, London, UK; 3 Population Health Improvement United Kingdom (PHI-UK), London, UK; 4 Lambeth Vocational Services, South London & Maudsley NHS Trust, London, UK; 5 Independent Researcher, London, UK; 6 Black Thrive Global,, Culturally Appropriate Peer Support and Advocacy Service (CAPSA), London, UK; 7 Care Policy and Evaluation Centre (CPEC), London School of Economics and Political Science (LSE), London, UK; 8 Division of Psychiatry, University College London (UCL), London, UK; 9 ESRC Centre for Society and Mental Health (CSMH) Lived Experience Advisory Board (LEAB), King’s College London, London, UK; 10 Department of Psychological Medicine, Institute of Psychiatry, Psychology & Neuroscience (IoPPN), London, UK

**Keywords:** Social inclusion, Social and economic adversity, Social interventions, Stratified analyses, Inequalities

## Abstract

**Purpose:**

People living with mental ill-health experience social and economic disadvantages, which contribute to poor outcomes and limit effectiveness of treatments. Interventions to improve social and economic circumstances have been developed, however, little is known about whether these interventions are effective for the most marginalised and disadvantaged groups, and those most in need of support.

**Method:**

We conducted a systematic review in line with a pre-defined protocol to identify interventions to improve the social and economic circumstances of people experiencing mental ill-health. We included relevant records from two previous systematic reviews and updated their searches across four databases. We synthesised the intervention domains and locations of research, participant characteristics, and if effectiveness varied by participant gender, socioeconomic position, and race or ethnicity, and related indicators. We worked in partnership with an advisory board including those with relevant lived experience to conduct this work.

**Results:**

We identified 266 relevant studies across 34 countries. Certain intervention domains were better researched than others (e.g. housing and employment vs. debt and social security advice). Participant characteristics were poorly reported resulting in a limited understanding of inclusiveness and generalisability of research. Only 8% of papers reported any stratified results and statistical reporting standards were poor, limiting our ability to determine what works for whom. Results from 4 RCTs indicated that interventions are less effective for those in lower socioeconomic groups.

**Conclusion:**

Improved reporting and representation of marginalised groups, stratified analyses of intervention data, and replication of results is needed to confidently draw conclusions about what works for whom in this field.

**Supplementary Information:**

The online version contains supplementary material available at 10.1007/s00127-025-02984-3.

## Introduction

People who experience mental ill-health are, compared with the general population, typically more disadvantaged across multiple domains, including education, employment [1, 2], housing stability and quality [3–5], income and finances [6, 7], and social isolation [8, 9]. Further, those with severe mental ill-health (e.g. psychosis) are more likely to have been exposed, over the life course, to violence, trauma, and discrimination [10–12]. These adversities increase risk of onset of mental ill-health and subsequent poor outcomes [1, 8, 13], contributing to an entrenched cycle of poor mental health and social exclusion. Furthermore, access to, and the effectiveness of, psychological and pharmacological treatment varies by indicators of socioeconomic position, thereby maintaining and widening inequalities in outcomes [14].

This relationship between social adversity and treatment outcomes highlights the need for mental health services to directly address the social and economic conditions of people with mental ill-health. Individuals with severe mental ill-health living in deprivation report significant unmet social needs [15], and failure of services to respond to such needs may contribute to lack of trust in services. This is particularly relevant to racial and ethnic inequalities in mental health systems. For example, in the UK, Black people with severe mental ill-health are more likely than people with severe mental ill-health from other ethnic groups to experience social and economic adversity [16–18] and this is further exacerbated by pervasive inequalities in access to mental health care [19–21]. Therefore, the relationship between social adversity and poorer treatment outcomes [14] means the failure of services to address social needs disproportionally disadvantages people from Black minoritised groups. Further dimensions of marginalisation within mental health care include social class, gender, and comorbid physical health and substance use problems, whereby the most socially excluded typically face multiple disadvantages and occupy multiple marginalised statuses [22, 23].

Interventions have been developed to interrupt this cycle of disadvantage by improving the social and economic circumstances of people with mental ill-health. Two recent systematic reviews synthesised this work [24, 25], finding consistent evidence that Housing First (HF) and Individual Placement and Support (IPS) were effective in addressing housing and employment needs, especially for people with severe mental ill-health. Additionally, some studies indicated that family psychoeducation interventions and supported socialisation interventions were effective in improving social circumstances. Both reviews highlighted the lack of research in other domains, especially related to finances (e.g., debt and social security).

An important but neglected aspect of this research concerns which interventions work for whom and in what contexts. At present, it is unclear to what extent studies are inclusive of the most marginalised groups and those facing multiple forms of adversity, within the already highly vulnerable group of people experiencing mental ill-health, or indeed whether studies report the characteristics of their samples in sufficient detail to assess this. As the key aim of social interventions is to improve outcomes for those experiencing social and economic adversity, it is essential that they are effective in supporting those who experience the highest level of need, otherwise they risk maintaining or exacerbating systemic inequalities.

## Aims

We conducted a systematic review to:

- i.Map the domains and contexts of research testing social interventions for people living with mental ill-health (severe mental illness and/or common mental disorders);
- ii.Summarise the gender, ethnicity and socioeconomic position of the participants recruited within these studies.
- iii.Assess how effectiveness of interventions varies according to these characteristics.

## Methods

We conducted a two-staged systematic review in line with a pre-established review protocol. This study was delivered in partnership with an advisory board comprising people with lived experience of mental ill-health, service providers, third-sector workers, and academics, recruited via the ESRC Centre for Society and Mental Health, networks local to South London including community organisations (e.g. Black Thrive), and services within South London and Maudsley NHS Foundation Trust. The research team and advisory board met regularly and made joint decisions about the research methodology, data analysis, and write-up, (Advisory board: co-authors MB, AC, GD, SL, BLE, CL). This review was conducted as part of a broader research program which additionally sought to identify targeted interventions designed to address social and/or economic needs in people living with mental ill-health from marginalised communities [26].

### Study selection and data extraction

We first identified studies included in two recent reviews [24, 25]. Both reviews included research detailing interventions which were designed to improve social and economic outcomes in adults with mental ill-health (see SI Table 1 for full inclusion criteria).

We then updated these reviews to identify literature published between January 2020 and February 2024, searching MEDLINE, Web of Science, PsycINFO, and CENTRAL (see SI for full inclusion criteria and search strategies). In line with the previous reviews, our inclusion criteria focused on non-pharmacological interventions designed to improve social or economic circumstances of adults with a severe mental illness or common mental disorder in any one of the following domains: housing/homelessness; money and basic needs; work and education; social isolation and connectedness; family, intimate and caring relationships; victimisation and exploitation; offending; rights, inclusion and citizenship (see SI Table 2 for full details). As the inclusion criteria for the two previous reviews varied slightly, we adopted the more inclusive approach where they conflicted (see SI Table 1&2 for discrepancies). Barnett et al. (2022) included systematic reviews (published from database inception- February 2020) and randomised controlled trials (RCTs) (published from 2000—August 2020); whereas Killaspy et al. (2022) included any peer-reviewed paper reporting primary empirical data published between January 2016 and July 2020. The updated search included records from July 2020-February 2024. We performed study selection for the updated search in duplicate (HB, AG, HW, ZA) at the title/abstract and the full-text screening stages.

We designed a fit-for-purpose data extraction sheet informed by data extracted in Barnett et al. (2022) with additional extraction of detail on socioeconomic and sociodemographic participant data relevant to our research question (see SI for further details). Individual researchers completed data extraction which was checked by a second researcher (HB, AG, HW, ZA, DL). Conflicts at all stages were resolved through team discussion.

### Quality appraisal

We extracted quality appraisals for studies included in the two previous reviews (i.e., Killaspy et al. (2022) used the Kmet [27]; Barnett et al. (2022) used the Cochrane Risk of Bias tool for RCTs [28]). For studies identified in the updated search, we used the Kmet quality assessment checklist as this could be applied to both quantitative and qualitative studies. A random proportion (10%) of quality appraisals were conducted by a second researcher (HB, AG, HW, ZA).

### Data synthesis

For data synthesis, we first summarised participant characteristics (diagnoses, sex/gender, race/ethnicity and socioeconomic position), country of research, and domain of interventions categorised into nine different domains broadly in line with classification frameworks [29]. Where data were not available on ethnicity or race, we extracted and synthesised any data on other related concepts including nationality, immigration status, heritage and indigeneity. This was necessary as countries have varying legal frameworks regarding such data (for example reporting data relating to ethnicity or race is not permitted in France).

Estimating inclusion of marginalised groups with respect to race and ethnicity is complex not least due to variations in such legal frameworks, conceptualisations of such social constructs, and structural racism within health research [30, 30–32]. Language and understanding related to these concepts are continually changing and it is crucial for researchers to keep these concepts under review while researching health inequalities [33]. In this paper, we report race and/or ethnicity data from individual studies using the language used in the respective studies, we also follow this for reporting of sex/gender. We note it was largely unclear whether such data pertained to participants’ self-ascribed identities or researcher observations.

We narratively synthesised studies reporting stratified analyses to assess whether effectiveness of interventions on social or economic inclusion outcomes varied by gender, ethnicity/race, or socioeconomic status and related indicators. For RCTs, we included any study where authors had assessed interaction effects between treatment condition and either sex/gender, ethnicity/race, or socioeconomic status; or conducted subgroup analyses or responder analyses based on these sociodemographic categories. For non-RCTs, we considered any study where authors assessed whether the intervention-related changes in social or economic inclusion outcomes varied based on these sociodemographic categories, including any subgroup analyses. We also synthesised studies that tested interventions developed for broader populations but reported results only concerning any one of these sociodemographic subgroups (e.g. women). We supplemented our synthesis with a separate summary of results of studies exclusively including participants with a psychotic disorder (see SI).

## Results

### Study characteristics

**Fig. 1:**
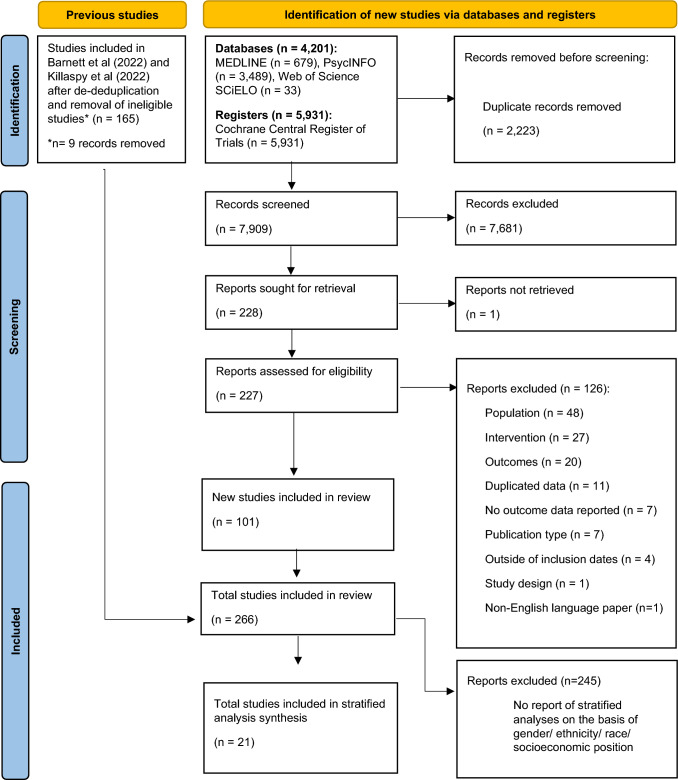
A PRISMA diagram demonstrating the flow of studies in the review

We included 165 studies from Barnett et al. (2022) and Killaspy et al. (2022) after de-duplication (n = 8) and removal of one meta-analysis. The updated database search identified a further 101 relevant records; therefore, 266 papers were included (See Fig. 1 for PRISMA and SI Table 3 for summary of study characteristics). For the studies identified in the updated search, Kmet quality appraisal scores ranged from 69%−100% for quantitative studies, and 65%−95% for qualitative studies. 

#### Intervention domains

**Fig. 2:**
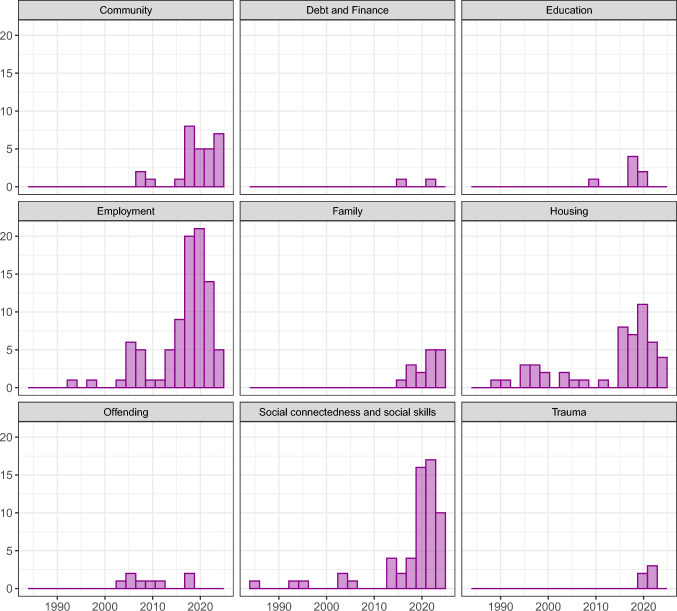
Histograms of primary life domains addressed by social intervention research over time. NB. Barnett et al. (2022) included systematic reviews (published from database inception − February 2020) and randomised controlled trials (RCTs) (published from 2000 − August 2020); whereas Killaspy et al. (2022) included any peer − reviewed paper reporting primary empirical data published between January 2016 and July 2020. The updated search included records from July 2020 − February 2024

Interventions addressed: Employment (n = 90; 34%), Social connectedness and social skills (n = 65; 24%), Housing (n = 50; 19%), Community support (n = 31; 11%), Family (n = 18; 7%), Education (n = 11; 4%), Offending (n = 9; 3%), Debt and Finance (n = 2; 1%), and Trauma and Victimisation (n = 5; 1%), where some addressed multiple domains (n = 15) (see SI Table 4 for examples of interventions in each domain). Research attention has increased overtime particularly within employment, housing and social connection domains (see Fig. 2).

#### Geographical location 

The included studies were conducted across 34 countries—the majority in the USA (n = 98) (See SI for heat map (Fig. 1) and full list;). Most studies were conducted within an urban setting (65%; both urban–rural: 9%; rural: 2%; unknown: 24%).

### Participant characteristics

No studies reported data on intersecting dimensions of marginalisation. 

#### Gender 

Most studies reported a male/female dichotomy only: 10 of the 266 studies (4%) reported on inclusion of individuals who identified as non-binary, transgender, gender-free or ‘other’. Fewer women were involved in research than men; the average inclusion of women was 43% (SD = 22%), compared with 58% for men (SD = 22%). 

#### Ethnicity, race, migration status, nationality, indigeneity, heritage

We found a range of relevant indicators were used in included studies, including ethnicity, race, nationality, migration status, indigeneity, and heritage.

Overall, 126 studies (47%) did not include any data on ethnicity, race, nationality, migration status, indigeneity, heritage or related indicators. For the 140 studies (53%) that did report on such data, there was variation in methods used. For example, research in some countries (e.g. France) was restricted to reporting nationality according to a binary (i.e. French national/not); in the UK and USA, ethnicity and race statistics were typically reported according to pre-defined census categories. Studies in the UK focused on the concept of ethnicity; studies in the USA typically reported statistics categorised by racial and/or ethnic groups.

In the UK and USA, ethnicity and/or race were reported by 104 of the 119 (87%) studies conducted in these countries. We were able to calculate pooled statistics for representation of ethnic and racial groups in these countries as most studies reported data according to consistent categories (see Tables 1 and 2). However, these pooled statistics are only based on studies which report this data, and are therefore likely to be inflated estimates, as studies which do not include any given ethnic group are more likely not to report any data. For example, only seven of all the included studies conducted in the UK (n = 21) report including any Black participants and therefore it is possible that the remainder did not include any Black participants (see Table 1).

**Table 1:** A summary of race and ethnicity representation across studies conducted in the United Kingdom (n = 21)

	Asian or Asian British	Black, Black British, Caribbean or African	Mixed or multiple ethnic groups	White (incl. Roma)	Other ethnic group (Arab/any other)	Unknown/rather not say
N studies reporting	6	7	5	13	9	2
Mean (%)	9.04	28.04	10.41	70.85	12.59	0.63
SD (%)	9.06	22.93	11.14	24.24	14.67	0.53
Range	1.75–26.5	6–70.97	1.5–29.03	17.4–100	0.75–50	0.25–1
IQR	5.5	20.95	8.7	26	6	0.36
Median	6	18	8	72.2	10	0–0.62

These statistics are calculated only from studies reporting data in these respective census categories. Studies not reporting data were excluded from these calculations. IQR = interquartile range; SD = Standard Deviation

**Table 2:** A summary of race and ethnicity representation in studies conducted in the United States of America (n = 98)

Race								Ethnicity					
	White/Caucasian	Black or African American	American Indian and Alaska Native	Asian	Asian/Pacific Islander	Native Hawaiian and Other Pacific Islander	Two or more races		Hispanic or Latino	White alone, non Hispanic or Latino	Black non-Hispanic	Non-Hispanic	Race/ethnicity unknown/other
N studies reporting	78	69	8	19	3	2	14		53	5	2	6	42
Mean (%)	47.00	42.21	3.85	3.64	2.89	3.5	11.52		13.43	65.62	35.55	84.32	19.13
SD (%)	20.59	23.21	2.58	4.97	3.21	0.71	11.70		11.13	28.04	7.71	8.33	20.34
Range	10.34–94.6	2.5–86.2	1–9.46	0–21	1–6.6	3–4	0.5–38		0–53	27–97	30.1–41	76–95.9	0.01–81
IQR	34.90	40	1.84	1.96	2.80	0.5	11.26		15.46	35.6	5.45	11.99	25
Median	49	41	3.5	2.7	1.08	3.5	8.95		12.5	64.7	35.55	81.27	10

These statistics are calculated only from studies reporting data in these respective census categories. Studies not reporting data were excluded from these calculations

It is difficult to assess inclusion of specific groups in countries beyond the UK and USA, due to lack of consistent reporting. Of the 149 studies conducted elsewhere, 41 (28%) reported data on ethnicity, nationality, and indigeneity (or related indicators). Many of these studies reporting such data were conducted in Canada (n = 17), and they mostly (n = 9/17) reported this data according to binary categorisations (53%) (e.g. “White” v “non-White”). There were 21 studies conducted beyond Canada, the UK and the USA that reported relevant data, and 17 (81%) of these reported this data according to a binary. We note one multisite study contributed multiple times to these counts, due to relevance to multiple countries, but did not report any relevant data. Due to these limitations in the data, we crudely estimated the inclusion of people from minority groups based on ethnicity, race, nationality, heritage, indigeneity and related factors all together in research in each country (see SI Table 5). 

#### Socioeconomic position

Indicators of socioeconomic position (including social class, occupation, tenure, financial situation, and education [34] were reported in 208 studies (78%). There was considerable heterogeneity in which indicators were used and studies often reported more than one indicator; the most common proxy was education (n = 148), followed by employment or vocational status (n = 65), homelessness (current/lifetime) (n = 33), income (n = 22), receipt of welfare benefits (n = 20), living status (i.e. owning home, private rental, social housing etc.) (n = 11), geography-specific socioeconomic stratum (n = 4), debt (n = 2), neighbourhood factors including the UK Index of Multiple Deprivation (n = 2), availability of food each day (n = 1), parental socioeconomic status (n = 1), housing stability (n = 1), and savings (n = 1). Due to the heterogeneity of the socioeconomic indicators used, and the variation in upper/lower bounds of socioeconomic disadvantage across geographies, it was not possible to define and assess inclusion of socioeconomically marginalised groups in a meaningful way across studies, and so we were not able to provide pooled summary statistics regarding the inclusion of the most socioeconomically marginalised groups here. 

#### Dual-diagnosis and comorbid physical health problems

Fifty-four studies (20%) reported substance use or abuse (current and/or lifetime) as an exclusion criterion to participation. Where the proportions with current substance abuse or dependence were reported (n = 69), this ranged from 1–100% (mean = 56%, SD = 32%). Comorbid physical ill-health data were reported less frequently (n = 27) and five studies excluded participants on this basis.

### Stratified analyses

Very few papers (n = 20; 8%) reported stratified results by gender, race and/or ethnicity, or socioeconomic position. Some studies reported multiple stratified analyses (29 analyses were reported between the 20 studies in total). No studies stratified results by multiple intersecting dimensions of marginalisation (e.g. women from low-income backgrounds). Most analyses were conducted on data from RCTs (n = 16) and many exclusively included people with severe mental illness (n = 13) (see Table 3 and SI Table 6 for further information on each study).

**Table 3:** Characteristics and results of studies conducting stratified analyses on the basis of gender, ethnicity/race/Indigeneity, and socio-economic indicators

Author, date	Country	Study design	Sample size	Ethnicity/Race/Nationality/minority status (%)	Gender (%)	Diagnoses (SMI, CMD)	Intervention domain	Quality Score)	Dimension of stratified analyses	Report sufficient data (Yes/No) Stratified analyses results	Main effect relevant to stratified analyses
Brown et al., 2016 k	USA	Retrospective pre-post analysis	182	56% White; 25% Black; 7% Asian/Pacific Islander; 13% Native American, Latino/a or multiethnic	74% Male; 26% Female	SMI	Housing	Kmet: 91 (Strong quality)	Previous homelessness	No Group x pre-housing days homeless interaction on homelessness outcome: F(1, 171) = 0.77; d = 0.14, Mean square = 14.28; p = 0.38; no further information provided	HF residents spent fewer days homeless in the post-housing year than the comparison group: F(1,171) = 87.05, p <.001, d = 1.44
Caplan et al., 2023u	Canada	RCT	43 parent–child dyads	NR	63% Male; 37% Female	CMD, SMI	Housing	Kmet:85 (Strong quality)	Indigeneity	Yes % reporting improvements in relationships with children: Indigenous parents—HF = 62% (n = 8/13); TAU = 13% (n = 1/8) Non-Indigenous parents—HF = 36% (n = 5/14); TAU = 25% (n = 2/8)	More parents reported positive changes in HF (48%; 13/27) v TAU (21%; 3/14)
Castelein et al., 2008b	Netherlands	RCT	106	NR	66% Male; 34% Female	SMI*	Community	High Risk of Bias	Gender	No	
Christensen et al., 2021u	Denmark	RCT	720	NR	62% Male; 38% Female	SMI	Employment	Kmet: 96 (Strong quality)	Employment history Gender	Yes Interaction effects between previous work history and study condition on vocational recovery for the following study condition comparisons: Work history was a stronger predictor of vocational recovery in the SAU group (those who had at least 2 month paid job in the last 5 years were 2.58 times more likely to work/study in the 18-month follow-up period) than in the IPS or IPSE groups (where they were 1.72 and 1.64 times more likely, respectively) Work history interacting with study condition on vocational recovery: IPS vs SAU: OR = 0.67, CI = 0.32–1.40, p = 0.287 IPSE vs SAU:OR = 0.63, CI = 0.30–1.35, p = 0.236 IPSE vs IPS:OR = 0.95, CI = 0.45–1.99, p = 0.889 Interaction effects between gender (male) and study condition on vocational recovery: IPS vs SAU: OR = 1.17, CI = 0.56–2.46, p = 0.674 IPSE vs SAU: OR = 0.57, CI = 0.26–1.25, p = 0.164 IPSA vs IPS: OR = 0.49, CI = 0.22–1.06, p = 0.073	Participants in IPS and IPSE groups had 2.2 times higher odds of having worked or studied during the 18-month follow-up than in SAU (OR = 2.22 95% CI 1.62 − 3.05)
De Waal et al., 2019b	Netherlands	RCT	250	72% Dutch; 8% Other; 6% Surinamese; 6% European; 4% Moroccan; 2% Dutch Antilles	70% Male; 30% Female	CMD, SMI	Trauma	High Risk of Bias	Gender; Education	Yes – Education Proportion of participants achieving treatment response regarding violent victimisation: Middle/High education = 82.6%; Lower education = 67.3%; OR = 2.46, 95%CI = 1.26–4.83, p = 0.009	Proportion of participants achieving treatment response for violent victimisation: Control group = 54% Experimental group = 67.6% [OR = 1.78, 95% confidence interval (CI) = 1.02–3.11, P = 0.042]
Dubreucq et al., 2020u	France	Quasi-experimental	87	NR	80% Male; 20% Female	SMI*	Social connectedness	100 (Strong quality)	Gender; Education	No	
Elbogen et al., 2016b	USA	RCT	184	74% Non-White	81% Male; 19% Female	CMD, SMI	Debt, Financial	High Risk of Bias	Annual income	Yes Annual income was related to the number of $AFE skills used (taught be the intervention) (b =.57, p =.02) and to the odds of using versus not using a $AFE budget (odds ratio [OR] = 1.64, 95% confidence interval [CI] = 1.16–2.32, p =.005)	No main effect on social/economic inclusion outcomes (money-saving behaviour, employment, debt, homelessness) of $AFE intervention v control condition (no statistics reported); Secondary analysis: use of $AFE skills taught in the intervention was associated with more responsible spending vs control participants (B =.41, SE =.191, p =.035) less impulsive spending (B = -.05, SE =.019, p = 0.14) and increased vocational activity (B =.22, SE =.097, p = 0.26)
Goldfinger et al., 1999b	USA	RCT	303	41% African American	72% Male; 28% Female	SMI	Housing	High Risk of Bias	Gender; Ethnicity; Education	Yes – Ethnicity Mean ± SD days homeless during follow-up: Independent living: African American or Hispanic = 107 ± 26 vs White = 48 ± 25 Staffed group housing: African American or Hispanic = 51 ± 29 vs White = 36 ± 32 B = 0.22, p <.01	Mean days homeless: Independent living = 78 Staffed group housing = 43 B =.22, p <.05
Gutman et al., 2009b	USA	Quasi-experimental	38	39% Hispanic, 37% African American; 21% White	58% Male; 42% Female	CMD, SMI	Education	High Risk of Bias	Education (participant and parent)	No	
Herman et al., 2011b	USA	RCT	150	62% African American; 17% White; 15% Latino; 6% Other	71% Male; 29% Female	SMI*	Housing	High Risk of Bias	Gender	No	
Hui et al., 2023u	Hong Kong	RCT	360	100% Chinese	44% Male; 56% Female	SMI*	Social connectedness	100 (Strong quality)	Gender	No	
Kidd et al., 2021u	Canada	RCT	110	24% Black or Afro-Caribbean or African; 9% East Asian or Asian; 8% South Asian; 4% Latino or Hispanic; 7% Middle Eastern or Arabic; 7% Other; Non -Hispanic White or European 43%	62% Male; 38% Female	SMI*	Community	100 (Strong quality)	Gender	No	
Marder et al., 1996b	USA	RCT	80	69% Non-White	100% Male	SMI*	Social connectedness	High Risk of Bias	Ethnicity; Education	No	
Martin-Carrasco et al., 2016 k	Spain, Portugal	RCT	223	NR	24% Male; 76% Female	SMI*	Family	96 (Strong quality)	Gender; Education	Yes Reduction of care giver burden within experimental group association with: i) Gender (Coefficient (95%CI)): Female: 0.14 (−0.12–0.41) ii) Education (Coefficient (95%CI)): Primary education: reference level Secondary education: 1.79 (−4.12–7.70) College, university: 2.79(−4.33–9.90)	Reduction in caregiver burden was greater for the experimental group v control at 4 and 8 months since trial inception SMD [95%CI]: 4 months: 0.35 [−64 to –0.06] 8 months: 0.40 [−0.70 to –0.10]
Maru et al., 2021u	USA	RCT	166	62% White; 38% Non-White or Mixed race; 22% Latino or Hispanic	49% Male; 51% Female	CMD, SMI	Employment	92 (Strong quality)	Receipt of disability benefits	Yes Participants receiving disability benefits were less likely to be classified as a responder (employed at anytime during 12-month follow-up): 83% of non-responders vs 52% of responders, χ2(1) = 8.54, p =.004	Intent to treat analysis: % of participants in work in experimental v control = no significant different (no statistics results reported) % looking for work: experimental (78%) v control (65%), χ2 = 3.54,p =.06
McHugo et al., 2004b	USA	RCT	121	83% African American	48% Male; 52% Female	SMI	Housing	High Risk of Bias	Gender; Lifetime homelessness	Yes- Gender Mean proportion of time spent in stable housing during each 6-month assessment period: Integrated housing – Females: mean = 0.69, SD = 0.36; Males: mean = 0.70, SD = 0.33; Parallel housing – Females: mean = 0.70, SD = 0.37; Males: mean = 0.40, SD = 0.40 F(1,107) = 8.32, p = 0.005	Mean proportion of days in stable housing was higher in integrated v parallel housing conditions at: 6 months, mean(SD) parallel v integrated: 0.40(0.38) v 0.47(0.33) 12 months: 0.62(0.42) v 0.80(0.28) 18 months: 0.68(0.40) v 0.85 (0.27) Group: F = 5.99, p < 0.05, d = 0.51
O’Campo et al., 2023u	Canada	RCT – Secondary analysis	653	24% Aboriginal; 53% White; 23% ‘Ethno-racial’	100% Female	CMD, SMI	Housing	100 (Strong quality)	Gender	Yes All analyses relate to female subgroup of larger trial Mean percentage of days spent stably housed during follow-up: TAU: 74.8% (95%CI = 71.7–77.8%), HF: 37.9% (95%CI = 34.4–41.3%), p < 0.001 Odds of stable housing during follow-up: OR = 5.09, 95% CI = 4.08–6.35, p < 0.001 Mean change from baseline to 24 months in community functioning: TAU = 4.8 (95%CI = 3.6–6.0), HF = 3.8 (95%CI = 2.8–4.9); p = 0.236 Mean change from baseline to 24 months in psychological community integration: TAU = 2.0 (95%CI = 1.4–2.6), HF = 2.0 (95%CI = 1.5–2.4), p = 0.941 Rate ratio of physical community integration in the past month: TAU = 1.03 (95%CI = 0.92–1.14), HF = 0.97 (95%CI = 0.86,1.08), p = 0.439	Woman-only subgroup analyses are the focus of the paper
Rebergen et al., 2009b	Netherlands	RCT	240	NR	56% Male; 44% Female	CMD	Employment	High Risk of Bias	Gender	No	
Rossler et al., 2020b	Switzerland	RCT	116	NR	49% Male; 51% Female	CMD, SMI	Employment	High Risk of Bias	Gender	No	
Scanlan et al., 2019 k	Australia	Uncontrolled prospective study	97	NR	47% Male; 53% Female	SMI	Employment	83 (Strong quality)	Gender	Yes Proportion achieving employment outcome: Male = 41.3% (n = 19/46); Female = 52.6% (n = 29/51);χ2(1) = 2.34, p = 0.13 Employment duration, mean (SD) days: Male = 165 (144); Female: 143 (169);t(45) = 0.46, p = 0.66	49.5% (n = 48) participants enrolled in IPS programme gained employment; average employment duration = 151 days (SD = 159 days)
Swinkels et al., 2023u	Netherlands	RCT	102	40% White; 24% Black African or Caribbean; 17% Arabic or Northern African; 2% Asian; 18% Multiple ethnic groups	88% Male; 12% Female	SMI	Social connectedness, Offending	100 (Strong quality)	Gender	Yes Criminal behaviour in intervention v TAU: Male (n = 89): RR = 0.519, 95%CI = 0.203—1.330; Female (n = 13): RR = 13.885, 95%CI = 2.090—92.253	Intention-to-treat analyses: TAU participants reported 2.9 times more criminal behaviours on average over time than intervention participants (RR = 0.346, 95% CI 0.152 to 0.787, p = 0.011)

Each study was classified as comprising “Sufficient data” where they included effect size and associated data (e.g. mean scores by group) relevant to at least one stratified analyses on the basis of each indicator. * = studies exclusively including participants with psychosis spectrum diagnoses. u = identified in updated search; k = identified in Killaspy et al. (2022); b = identified in Barnett et al., (2022). RCT = Randomised Controlled Trial; SMI = Severe Mental Illness, CMD = Common Mental Disorder; NR = Not Reported. HF = Housing First; IPS = Individual Placement and Support; TAU = Treatment as Usual. QA kmet scores reported for studies included from the updated search and Killaspy et al., whereas Cochrane Risk of Bias scores are reported for studies included from Barnett et al., 2022, obtained via communication with the authors. See SI Table 6 for further details on stratified analyses

Many factors limit our ability to draw conclusions about variation in effectiveness of social interventions for the different groups examined. There was no replication of stratified analyses as each pertained to studies of different interventions and settings. Further, authors reported sufficient effect size data to interpret the results in less than half of stratified analyses (45%; 13 of 29); rather, if effects were found to be not ‘significant’, researchers often did not report any data. Nevertheless, some evidence indicated that effectiveness of interventions may vary for different groups. Evidence from 2 strong quality and 2 high risk of bias RCTs suggests that people from lower socioeconomic groups benefit less from some interventions. The extent to which outcomes vary by socioeconomic or sociodemographic group likely depends on the specific domains, designs and contexts of interventions. However, given the sparsity and limitations of existing evidence, it is not yet possible to investigate these patterns.

#### Gender

Fifteen studies (5%) stratified analyses by gender. This included one non-controlled prospective study of an employment intervention [35], one quasi-experimental controlled study of a social connectedness intervention [36], and 13 RCTs (see Table 3; SI Table 6). Of the 13 RCTs, 5 reported effect sizes data regarding the possible moderating effect of gender on the impact of the intervention. Two found a stronger effect for men [37, 38], one reported a subgroup analysis finding evidence for effectiveness in women [39], and two studies reported effects with confidence intervals indicating a range of possible of interpretations [40, 41].

For example in a strong quality-assessed, two-arm, multicentre RCT (n = 223), which tested the efficacy of a psychoeducational intervention programme designed to reduce caregiver burden for carers of people with schizophrenia [41], a subgroup analysis suggested that the intervention may be more effective for women (i.e., there was a moderate association with burden reduction among women but not men). However, data were statistically consistent with parameter values ranging from a considerable level of reduced risk to a considerable level of increased risk of burden reduction (Coefficient (95%CI): Female: 0.14 (−0.12–0.41)).

There was no replication within these 5 RCTs: they all tested different interventions across the domains of housing, employment, social connectedness and family relationships, and many suffered limitations such as small number of women included [38].

#### Minoritised ethnic groups and Indigenous peoples

We identified 3 studies (1%) that stratified analyses by ethnic group or related indicators. These included one qualitative study of a Housing First intervention in Canada [42], one RCT of independent vs. staffed group living for homeless people in the USA [43], and one RCT of social skills training for people with schizophrenia [44] (see Table 3; SI Table 6). This is too limited an evidence base to draw any conclusions about variation in effectiveness of social interventions by minoritised ethnic or racial group.

Nevertheless, the RCT of independent vs. staffed group living for homeless people (n = 303) did find that, at 18-month follow up, African American and Hispanic participants experienced on average 37 more days of homelessness than White participants, across both conditions (an average of 59 more days of homelessness in the independent living condition; and 15 more days in the staffed condition) [43]. This suggests poorer outcomes in both intervention arms for minoritised populations, but that this inequality may be reduced in staffed living interventions. However, this is a single study, rated as having a high risk of bias (see Sect."Quality appraisal"). The other RCT of social skills training reported that there was no ‘significant’ interaction effect by ethnic group, making it impossible to assess whether there was any variation in effect.

#### Socioeconomic position

Eleven studies (4%) stratified results by indicators of socioeconomic position (see Table 3; SI Table 6). Three of these were non-RCTs, examining interventions in the domains of housing [45], education [46], and social connectedness [36]. None of these non-RCT studies reported sufficient information on effect size and/or group means to interpret their results.

Evidence from 4 RCTs indicated that interventions were less effective for people in lower socioeconomic groups [47–50]. These interventions spanned the domains of victimisation, debt and finances, employment, and family relationships; and sample sizes ranged from 166 to 250. For example, in one study rated as strong quality (n = 166), responder analysis of a vocational peer support intervention found that participants receiving social security payments were less likely to be classified as responders, i.e. become employed at any time during the 12-month follow-up (those receiving social security payments comprised 83% of non-responders but 52% of responders) [49]. One of the remaining RCTs was rated as strong quality [50], but the remaining two were rated as having high risk of bias [47, 48].

One further RCT (n = 720) was inconclusive regarding whether an employment intervention was more effective for people in higher socioeconomic positions (as indicated by work history: having had a paid job for at least two months in the last five years), as data were statistically consistent with parameter values ranging from lower to higher likelihood of intervention success (e.g. OR = 0.67, CI = 0.32–1.40). However, although interventions may be less effective for those facing more challenging socioeconomic conditions, this strong quality RCT highlighted that this inequality is reduced in the intervention condition compared with treatment as usual: the impact of previous work history on vocational recovery outcomes was lower for participants receiving IPS compared with those receiving service as usual [40]. Three further RCTs reported stratified analyses. However, they did not report sufficient accompanying data to interpret their analyses [37, 43, 44].

## Discussion

In our review we found that interventions seeking to improve social and economic circumstances of people with mental ill-health rarely considered of the social contexts in which interventions are tested and the social groups included. When relevant information was included, variations in reporting and data missingness did not allow for a complete understanding of inclusion of marginalised groups. There was similarly a lack of data regarding people who experience multiple intersecting forms of marginalisation. In the rare instances where analyses were stratified by social group, some studies found that people from marginalised socioeconomic positions may benefit least. Overall, we found that the current body of research does not provide any substantive information on which interventions work for whom — and whether they are effective for the most marginalised within an already highly vulnerable group: people experiencing mental ill-health who face social and economic adversities.

We note there are extensive systemic barriers to designing, implementing and evaluating interventions and services which address social and economic challenges of people with mental ill health across the continuum of need, including the strong focus on medical intervention over social intervention in contemporary services, as well as lack of resource for social interventions [51, 52], which contextualise the lack of work we identified in this space.

There are some limitations that should be considered when interpreting our findings. Due to the slight discrepancy in search strategies between the two reviews we updated, coverage of the literature was less thorough for the period covered by the two previous reviews compared with the updated review period. For example, non-RCTs recruiting people with common mental disorder diagnoses would have been missed by the two previous reviews as Barnett et al. (2022) only included RCTs and Killaspy et al. (2022) only included studies of severe mental illnesses. Further, as we screened for samples with a diagnosed mental disorder or who had accessed mental health services, we may have missed social care studies not based on diagnostic frameworks. Related to this, as we restricted our search to articles published in peer-reviewed journals, we overlooked social interventions evaluated in the grey literature. Additionally, we only included English language papers and may have missed studies conducted in non-English-speaking countries.

### Social intervention research domains, contexts and participants

Most research we identified was conducted in the Global North, with most in the USA, and in urban settings. We observed a marked increase in research on interventions to improve social and economic circumstances since approximately 2010; however, this has clustered across a few specific domains. Most interventions focused on housing, employment, social connectedness and community participation. There are areas of social and economic need that are largely neglected, notably interventions addressing debt, finances, and victimisation. We did not identify any studies testing the impact of primary prevention interventions, such as Universal Basic Income, which would also address the socioeconomic needs of those with mental ill-health [53, 54]. More work is needed given that debt, poverty and need for social security are very high for people with mental ill-health and can impact recovery [18, 22, 55–57]. Equally, mental ill-health can impact a person’s ability to manage finances and navigate complex social security systems [58–61].

Data on ethnicity and associated concepts were often poorly or incompletely reported. Inadequate reporting sometimes stemmed from national policy and legal frameworks. Estimating representativeness of recruited samples was beyond the scope of this review, and would be highly complex given the lack of national audit data available on use of social interventions in mental health services, despite some local research [62]; as well as the sociodemographic variations in prevalence of mental ill-health and service use – where the discrepancy between these two factors is commonplace given that many people face barriers to accessing support. Nevertheless, our data highlights that intervention research may not be generalisable to contexts where the service users are not of majority White ethnicity: for example, White participants comprised the majority [70.85%) reported in UK samples, and as such may not apply to many services and contexts in the UK such as London where populations are more ethnically diverse.

Socioeconomic status and sex/gender were more consistently reported although only 4% of studies reported any data on non-binary genders. Fewer women were recruited to the included studies, which may in part reflect variations in the prevalence of mental health problems e.g., higher proportion of men experience psychosis; however, as with ethnicity, investigations of representativeness must also consider variations in prevalence of needs in the different social and economic domains between different genders, as well as use of social interventions.

Inclusion was also limited regarding people with co-occurring conditions, substance use and physical ill-health. Approximately 1 in 5 studies excluded participants with substance abuse or dependence problems, which limits our ability to draw conclusions about intervention effectiveness for some of the most vulnerable in society. For studies including such populations, average comorbidity with a substance use disorder was high (55%). Even fewer studies reported the prevalence of physical health conditions in their samples. This is problematic given interactions between such factors and mental ill-health, which worsen outcomes [63–65]. Further research is also required to understand the extent to which social intervention research has included people with neurodevelopmental conditions, such as autism, who are at high risk of developing mental ill-health [66], yet are marginalised within mental health care systems [67, 68] and experience heightened social exclusion [69].

We found current reporting standards in social intervention research prevent a nuanced understanding of how forms of marginalisation may impact outcomes. Poor reporting of sociodemographic characteristics precludes any consideration of variation by, and at the intersection of, marginalised groups. This is problematic given that mental health outcomes are worse for people in multiply marginalised groups [70]. This reflects the dominant conceptualisation of social adversities in mental health research, whereby problems are located within the individual, as distinct risk factors (e.g. socioeconomic status, ethnicity) without consideration of how such factors may interact, or of the processes and social structures contributing to poor outcomes [71]. Indeed, drawing on intersectionality theory, we emphasise that forms of marginalisation that are experienced together cannot be understood through separation into isolated entities [23]. Together, this calls for standardised, nuanced reporting processes of socioeconomic adversity in future studies.

### What works for whom?

Our synthesis shows that we do not know what social interventions work best for whom. Under 10% of studies investigated the effectiveness of interventions by socioeconomic and sociodemographic groups. No studies or analyses were replicated.

Only 45% (n = 13) of the stratified analyses reported adequate data for us to interpret their findings. Most inferred effectiveness based on statistical significance testing against arbitrary p-values (i.e. p ≤ 0.05), and did not report any data for analyses which were classed as “non-significant”. This approach of reporting results according to thresholds of significance is problematic and leads to misleading conclusions [72–75]. In addition to poor statistical reporting standards, the underrepresentation of marginalised groups we identified limits our ability to reach satisfactory conclusions about effectiveness. Increased sample sizes must be employed to increase precision and reduce uncertainty in inequalities research. One way to do so is to foster open science initiatives whereby anonymised trial datasets are made available to other researchers to harmonise with other datasets and test outcomes for specific groups across multiple studies. Equally, improved attempts to pre-specify a priori subgroups for subgroup analyses are needed, particularly given the persistent problem of trials reporting unplanned subgroup analyses after finding “statistically significant” effects [76].

Within this context, some evidence suggested that effectiveness of social interventions may be lower for people in lower socioeconomic groups. Most RCT studies reporting sufficient data (4 out of 5 RCT studies) found this, including interventions to prevent victimisation [47], assist with budgeting skills [48], provide vocational peer support [49], and reduce caregiver burden [50]. Further research is needed to firmly draw conclusions regarding socioeconomic position due to the limited number of studies.

The paucity of stratified analyses was most striking with respect to ethnicity. This is particularly problematic given the high inequalities in mental healthcare access and outcomes for minoritised ethnic groups [77–79]. Further, these studies were constrained to the domains of housing and social skills, but we know people with mental health problems from minoritised ethnic groups experience heightened adversity across other domains, including social isolation [17, 18] and unemployment [80], among others.

## Conclusions

In sum, we do not know whether the effectiveness of existing social interventions varies for different groups, although there was some indication that interventions risk reproducing existing inequalities due to lower effectiveness for those from more disadvantaged socioeconomic groups. The lack of stratified analyses prevents us from assessing whether findings from intervention research are translatable to local populations in practice. More nuanced research trials, open science efforts, and more representative recruitment practices are required.

## Supplementary Information

Below is the link to the electronic supplementary material.

Supplementary file1 (DOCX 426 KB)

## Data Availability

Data available upon reasonable request.
